# Hexavalent chromium ion removal from wastewater using novel nanocomposite based on the impregnation of zero-valent iron nanoparticles into polyurethane foam

**DOI:** 10.1038/s41598-024-55803-1

**Published:** 2024-03-05

**Authors:** Eman M. Saad, Mohammed F. Abd-Elhafiz, Eman M. Ahmed, Ahmad Abo Markeb

**Affiliations:** 1 Chemistry Department, Faculty of Science, Suez University, Suez, Egypt; 2 Chemistry Department, Faculty of Engineering, South Vally University, South Vally, Qena, Egypt; 3 Department, Faculty of Science, Assiut University, Assiut, Egypt

**Keywords:** Polyurethane foam, Hexavalent chromium removal, Zero-valent iron nanoparticles, Wastewater, Response surface methodology, Modelling, Environmental sciences, Chemistry, Materials science

## Abstract

In this study, we developed a novel nanocomposite, polyurethane foam impregnated with zero-valent iron nanoparticles (PU@nZVI), for the effective removal of chromium(VI) from various water sources. The characterization of nanocomposite (PU@nZVI) was performed by XRD, SEM–EDS, TEM and FT-IR techniques. Using the response surface methodology, we optimized the removal conditions, achieving an optimal pH of 2 and a dose of 0.5 g/L. The PU@nZVI demonstrated an excellent maximum adsorption capacity of 600.0 mg/g for Cr^6+^. The adsorption kinetics and isotherms were best described by the pseudo-second-order model and the Freundlich isotherm, respectively. Significantly, the nanocomposite removed 99.98% of Cr^6+^ from tap water, 96.81% from industrial effluent, and 94.57% from treated sewage wastewater. Furthermore, the PU@nZVI maintained its efficiency over five adsorption–desorption cycles, highlighting its reusability. These results suggest that the PU@nZVI nanocomposite is a highly efficient and sustainable option for chromium(VI) removal in water treatment applications.

## Introduction

Water is a vital natural supply for the improvement of life and human activities. Over the last decades, the scarcity of water and its quality have developed issues of significant concern^1^. High quantities of contaminated water have been continuously produced, specifically in industrialized and developed countries. In addition to the global scarcity of water, the discharge of contaminants with the potential to endanger both the environment and human beings into water bodies is the chief risk to the world’s freshwater resources^1–3^. The recovery of the quality of water is crucial to avoid higher levels of contamination, deal with the zero-release idea, and aid water reuse. Water management implementation is required to achieve the proper quality of wastewater effluent treatment plants^4,5^. Diverse grades of management will be needed according to the final usage of this reused water.

Releasing wastewater effluents containing heavy metals from many industries into water streams is one of the most significant ecosystem concerns^6^. For instance, the inorganic heavy metal contaminants have a physiological alarm^7–9^ owing to their hazards to human health and the aquatic environment^10,11^. For example, chromium (Cr^6+^) is harmful and toxic even at low concentrations due to its bioaccumulation and its capability to create active oxygen species in human and animal living cells^12–14^. Moreover, large amounts of effluents with high Cr^6+^ concentrations are yielded from many industries, such as leather tanning, textiles, glass, ceramic manufacturing, and electroplating. World Health Organization (WHO) legalized a maximal tolerable quantity of total Cr in drinking water as being 0.05 mg/L^15^. So, the high-efficiency removal of these metal ions is considered a dire need. Numerous methodologies have been reported for chromium minimization from contaminated water, including precipitation^16^, ion-exchange separation^17^, membrane filtration^16,18^, photocatalysis^16,19^, and adsorption^20,21^.

The adsorption technique has been studied as an economical, straightforward process that requires few chemical additives^6,22–24^. To date, Zero-valent iron nanoparticles (nZVI) have been considered as a Cr^6+^ capturing agent due to their significant reactivity, high specific surface area, and low cost. However, employment of ZVI nanoparticles alone is not sufficient for effective contaminants removal from aqueous solutions due to (1) their aggregation tendency, (2) their ability to form precipitates, and (3) their ability to interact with other components in the water environment^25^. These demerits can be avoided by incorporating nZVI particles onto a spongy media to improve its removal efficiency. Several sorbents impregnated by nZVI particles were utilized for capturing Cr^6+^ and have been testified as activated carbon (AC)^26^, chitosans^27^, biochar^28^, bentonite^29^, carbon nanotubes^30^, humus^31^, and silica^32^. Some of them exhibit low removal efficiencies and costly and tedious synthesis procedures. For instance, the activated carbon doped with nZVI (AC/nZVI) was used as a sorbent for Cr^6+^ removal with a sorption capacity of 25.00 mg/g^25^. The maximum adsorption capacity of activated carbon derived from wood was used to eliminate Cr^6+^ with an adsorption capacity of 241.55 mg/g^16^. Zeolite coated with magnetic nanoparticles was used to remove Cr^3+^ with a maximum removal capacity of 43*.*93 mg/g^33^.

Polyurethane (PU) Foam is a synthetic polymer that has a broad range of physical and chemical properties and many applications, owing to its excellent hardness, elasticity, high flexibility, and capability to resist extreme temperature and pH conditions^34^. Blends of polyamide have been given importance due to their imperfect pore-size conveyance, upgraded chemical inertness, and mechanical properties^35^. However, it has low ligand firmness, and its frame exhibited defective sorption. When mixed with other materials, it could enhance the responsive purposes in the network, and hence, it has been used effectively for the elimination of contaminants from wastewater^34^. Further, the recyclability of polyurethane foam can be quickly done after the desorption route of pollutants^34^.

Therefore, this study aims to (1) synthesize novel magnetic nanocomposites by impregnating the zero-valent iron nanoparticles into polyurethane foam (PU@nZVI), (2) to optimize the amount of the nZVI loaded into PU for better Cr^6+^ removal from wastewater as sorbent nanomaterial and hence to determine their properties, (3) to optimize the independent variables influencing the batch removal process of Cr^6+^, such as pH and dose of the adsorbent using response surface methodology (RSM), (4) to examine the effect of time, initial Cr^6+^ concentration and temperature on the adsorption process and hence estimate the adsorption mechanism, and (5) to evaluate the efficacy of the synthesized NC via the reusability, adsorption–desorption cycles, and their applications into wastewater treatment.

## Materials and methods

### Chemicals, reagents, and preparation of solutions

Potassium dichromate, diphenyl carbazide, sodium borohydride, iron(III) chloride hexahydrate, and solvents (acetone and ethanol) with purity higher than 99% were purchased from Sigma-Aldrich, Germany. Sodium hydroxide and mineral acids (HCl and HNO_3_) were purchased from Alfa Aesar, Germany. All solutions were prepared with ultra-pure water from the Milli-Q system (Millipore, USA). Details of the preparation of solutions are described in Supplementary Information (S1.1.).

### Synthesis of PU@nZVI NCs

The synthesis of PU@nZVI NCs was carried out in two steps, as described below.

1. Firstly, the nZVI was synthesized by the liquid phase method through the ferric ion reduction using sodium borohydride (NaBH_4_), as reported^36^. Typically, FeCl_3_⋅6H_2_O (1.0862 g) was dissolved in 200 mL of water/ethanol mixture (1/4, v/v). After that, 200 mL of Milli-Q water containing 0.7570 g of NaBH_4_ was added dropwise to the solution of iron (III) chloride until a black color of the solid ZVI NPs was formed. Then, the mixture was vigorously stirred for 20 min. Finally, the nZVI were dried in an oven at 50 ℃ overnight after washing three times with Milli-Q water and ethanol using magnetic decantation.
2. Secondly, the magnetic PU@nZVI NCs were prepared by impregnation of the dried ZVI NPs into the PU support. Different amounts of nZVI (25, 75, 150, and 300 mg) were briefly sonicated in 100 mL of ethanol for 2 h at room temperature. Then, one gram of PU was soaked in each nZVI suspension for 30 min, followed by isolation of the PU@nZVI NCs and drying at 50 °C for 12 h. The samples were coded as S25, S75, S150, and S300.

### Characterization of the nanocomposites

The morphology, elemental composition, and elemental mapping of PU@nZVI NCs were measured using a Field Emission Scanning Electron Microscope (FE-SEM), Zeiss SEM Ultra 60.5 kV, with an Energy Dispersive X-ray spectrometer (EDS), Zeiss, Germany. Transmission Electron Microscope (TEM), JEOL JEM 1400 (120 kV), Germany, was used for morphology and size determination of the nanoparticles. Functional groups of PU@nZVI NCs were investigated using Fourier Transform Infrared Red (FTIR) spectroscopy, Nicolet Nexus 470 FTIR instrument, USA, with standard KBr method. The crystallinity phases of PU@nZVI NCs were monitored using powdered X-ray diffraction (XRD), Philips 1700 version diffractometer with H. T. P. W 1730/104 KVA. The anode was Cu Kα (λ = 1.54180 Å). The amount of nZVI loaded on PU was analyzed by Atomic Absorption Spectroscopy (AAS), Contra AA 700, Analytik Jena, Germany, after digestion of the NCs in HNO_3_ solution (10%) for 24 h.

### Adsorption testing

The sorption of Cr^6+^ on PU@nZVI NCs was carried out using batch experiments. In this work, the efficacy of the synthesized NCs was first assessed by studying the amount of nZVI loaded onto PU support. Typically, 25 mg of S25, S75, S150, and S300 NCs were added separately into a bottle containing 25 mL of Cr^6+^ solution (10 mg/L) and each flask was shaken at 25 °C and 180 rpm using an incubating shaker, JSR Korea. To investigate the impact of the initial concentration of Cr^6+^ ion on the sorption by the PU@nZVI NCs, different Cr^6+^ concentrations, 0.5–700 mg/L, were used after optimization using a central composite design as described below. Next, the equilibrium time was determined by taking a 25 mL solution of 100 mg/L Cr^6+^ containing S300 adsorbent, and the mixture was agitated at selected intervals of time ranging from 10 to 720 min. Thermodynamic parameters were estimated by studying the effect of temperature ranging from 25 to 40 ℃. Depending on the experiment, the solution pH was adjusted by NaOH and/ or HCl, both at 0.1 M, using pH meter, Horiba, USA. The residual of metal ions was analyzed by the standard test method at λ_max_ = 540 nm^37^. Consequently, the removal efficacy (%), the adsorbed amount of Cr^6+^ per unit mass of PU@nZVI NCs at equilibrium, Q_e_ (mg/g), and time t, Q_t_ (mg /g) were calculated using the equations described in the Supplementary Information (S1.2.).

Also, detailed information regarding models used in isotherms, kinetic studies, and equations used to estimate the thermodynamic parameters to fit the experimental data were presented in the Supplementary Information (S1.3.).

### Optimization parameters of Cr^6+^removal

#### Central composite design

Significant factors were determined using an experimental design to invest time and cost. To optimize the Cr^6+^ removal, a central composite design (CCD) model was employed. Two independent variables, pH and amount of PU@nZVI NCs, were investigated. The design table and quadratic equation for model fitting to obtain the optimum conditions for adsorption were shown in the Supplementary information (S1.4.).

#### Response surface methodology and statistical analysis

The response surface methodology (RSM) was performed to determine the relationship between groups of test factors to determine the optimal conditions. So, the experimental statistics obtained from CCD were analyzed by RSM, and then the mathematical model was determined. The quadratic model is the most commonly used, a 2nd-order polynomial relation fitting the experimental data by Equation S9 in the Supplementary Information.

The analysis of variance (ANOVA) was employed to justify the significance and competence of the developed regression model. The competence of the RSM model was validated by calculating the correlation coefficients (R^2^), adequate precision, and lack of fit^38,39^.

#### Regeneration and reusability experiments

The reusability study was performed by sorbing 10 mg/L of Cr^6+^ with S300 NCs under the optimum conditions. Next, the PU@nZVI NCs were reused by magnetic separation and washing with deionized water to eliminate the non-adsorbed Cr^6+^. Then, the NCs were exposed to three different eluents of HCl, HNO_3,_ and NaCl solutions (0.01 M) for two h and 150 rpm at 25 ℃. After that, the recycled adsorbents were reused to adsorb fresh Cr^6+^ solution (10 mg/L) under the same adsorption conditions. The efficacy of the regeneration was assessed by repeating the adsorption/desorption study of Cr^6+^ for five cycles.

## Results and discussion

### Influence of loaded nZVI on PU

Table 1 displays the iron (Fe) concentration in the loading of S25, S75, S150, and S300 (milligrams of Fe per gram PU foam), which yields 5.614 ± 0.002, 9.030 ± 0.001, 22.564 ± 0.003 and 36.125 ± 0.003 mg Fe/g PU, respectively. Accordingly, the higher loading capacity (36.125 ± 0.003 mg Fe/g PU) was chosen for further tests.

**Table 1:** Concentration of iron in polyurethane foam NCs.

Sample code	ZVI concentration, mg_Fe_/g_Foam_
S25	5.614 ± 0.002
S75	9.030 ± 0.001
S150	22.564 ± 0.003
S300	36.125 ± 0.003

### Characterization of PU@nZVI NCs

**Figure 1:**
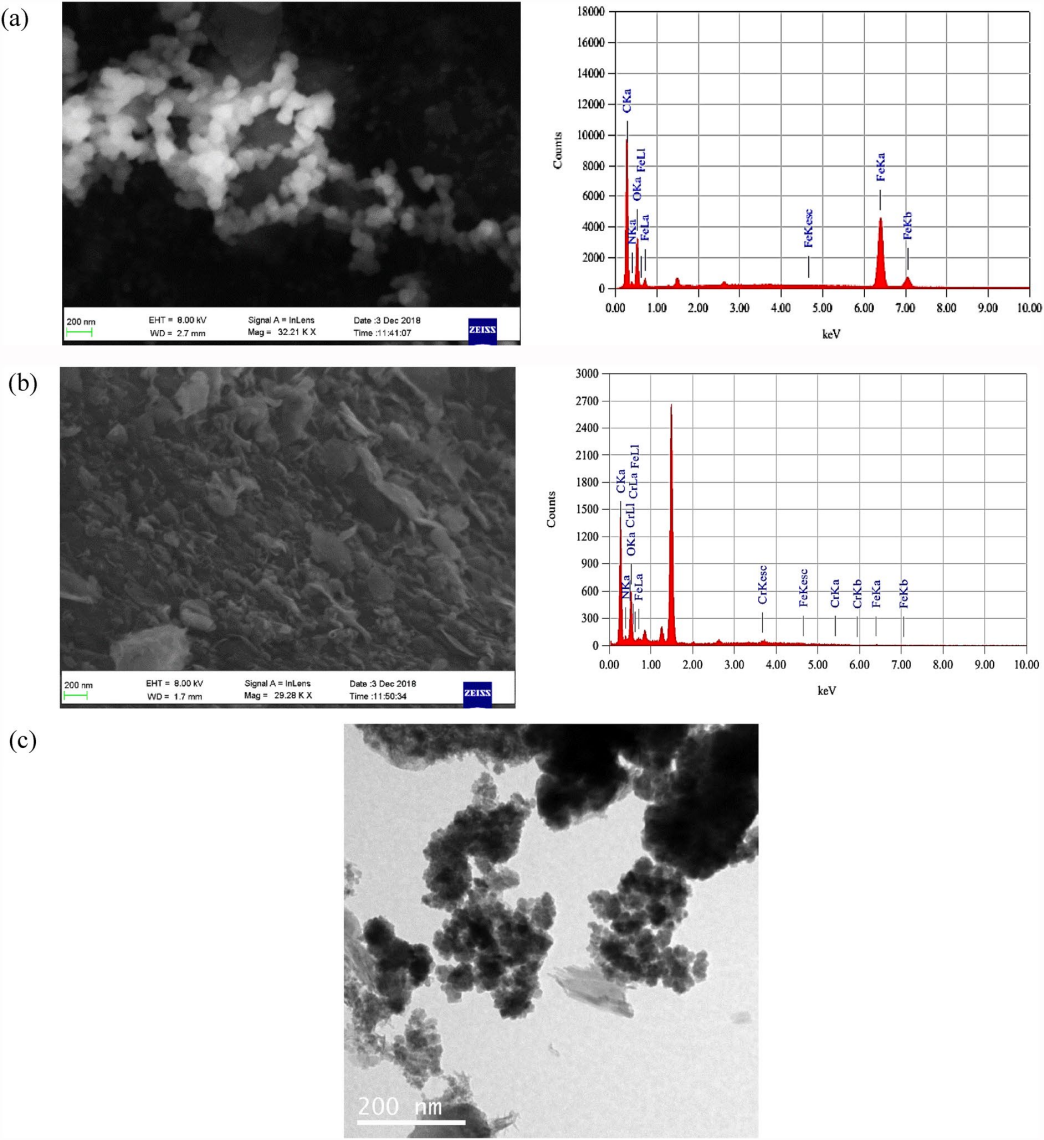
SEM image with EDX of nZVI synthesized loading in Polyurethane Foam before (**a**) after (**b**) Cr^6+^ removing and TEM image of zero-valent iron in the PU@nZVI (**c**).

Scanning electron microscope (SEM) of the PU@nZVI NC was conducted to identify its morphological structure. As illustrated in Fig. 1, the SEM images of PU@nZNI NCs, before and after adsorption of the Cr^6+^, exhibited a smooth surface (Fig. 1a) and changed to rugged and multi-hole morphology to a large extent (Fig. 1b), thus forming large numbers of micropores on the surface^36,40^. In addition, the nZVI particles were sphere-shaped nanoparticles (NPs)^41^ with an average diameter of 200 nm and filled the pores of PU Foam. This observation suggests that the nZVI NPs can penetrate and occupy the pore spaces within the foam material, indicating their potential for efficient adsorption and catalytic activities. The EDX pattern of loaded PU@nZVI with Cr^6+^ exhibited signals of Cr besides the other signals of Fe, C, N, and O observed in the EDX pattern of unloaded PU@nZVI NCs, which represents the adsorption of Cr^6+^ onto PU@nZVI NCs (Fig. 1a,b). Moreover, the morphology and size of the nZVI particles were illustrated in Fig. 1c. It was found that the particle size of the zero-valent iron nanoparticles is 11.09 ± 1.98 nm. Also, the TEM image of the nZVI exhibited a spherical shape, which is in agreement with the reported study^42^.

**Figure 2:**
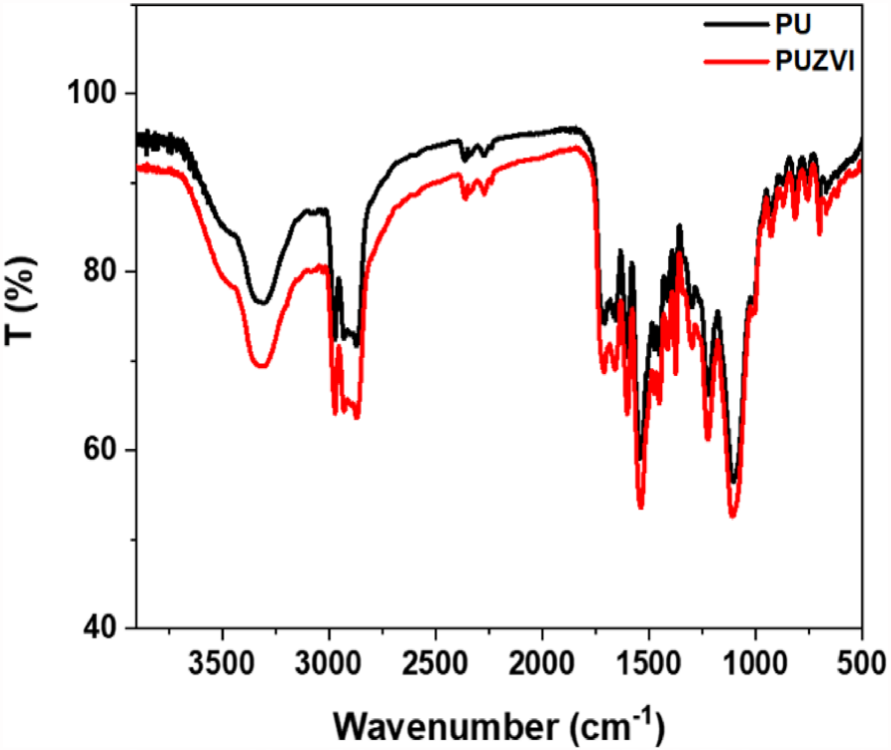
FT-IR spectra of free PU and its PU@ nZVI NCs.

As illustrated in Fig. 2, the FTIR spectra of PU and PU@nZVI NCs exhibit absorption bands around 3316 cm^−1^ and 3400 cm^−1^, representing the stretching vibrations (–N–H) and (OH) of adsorbed water, respectively^43^. The band at 1730 cm^−1^ was assigned to ν (–C=O) group, indicating the polyurethane moiety's presence. In addition, the bands at 2868 cm^−1^ and 2970 cm^−1^ can be attributed to the symmetric and asymmetric stretching of -CH bonds^44,45^.

**Figure 3:**
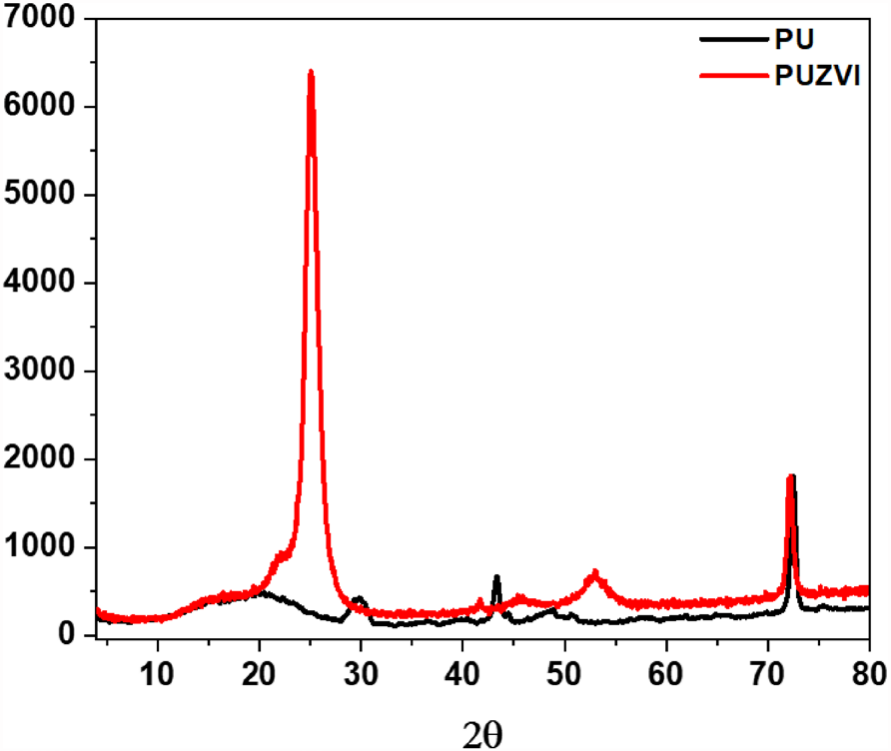
XRD pattern of the produced nZVI loaded into Polyurethane Foam.

A powder X-ray diffraction (XRD) pattern was employed to characterize the crystallinity degree of PU and the PU loaded with ZVI NPs. As demonstrated in Fig. 3, the XRD pattern of the PU@nZVI NC displays two characteristic sharp peaks at 2θ of 25°, corresponding to the zero-valent iron^41^. It corresponds to the (111) plane of the crystal lattice, as indicated by the reference database Fe-04-008-1610. In addition, the appearance of a characteristic broad peak at 2θ of 18° and two weak peaks at 2θ of 30° and 43° relates to the Polyurethane Foam sample^46^.

### Effect of nZVI loaded into foam

**Figure 4:**
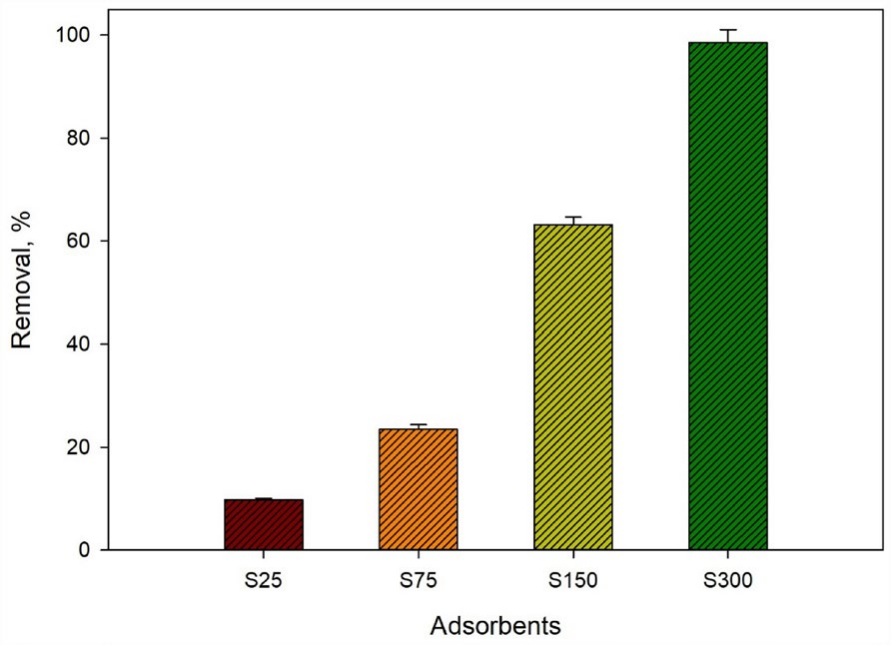
Effect of nZVI amounts loaded into PU Foam on the removal percentage of Cr^6+^.

Different amounts of zero-valent iron NPs (nZVI) were loaded onto PU Foam to investigate their removal efficiency towards removing Cr^6+^. The results, depicted in Fig. 4, exhibit the removal efficiency of Cr^6+^ increasing with increasing the amount of nZVI on PU foam, and the maximal removal efficacy was acquired at 36.125 ± 0.003 mg_Fe_/g_PU_ for the code S300. Therefore, the NC labeled with S300 was selected for the subsequent adsorption experiments and characterization.

### Experimental design

The experimental design matrix provides insights into the validities of dosage and pH on the removal percentages and adsorption capacities. The results demonstrated variations in performance based on the specific combination of variables, emphasizing the importance of optimizing these parameters for achieving high removal efficiency in the studied system^47^.

**Figure 5:**
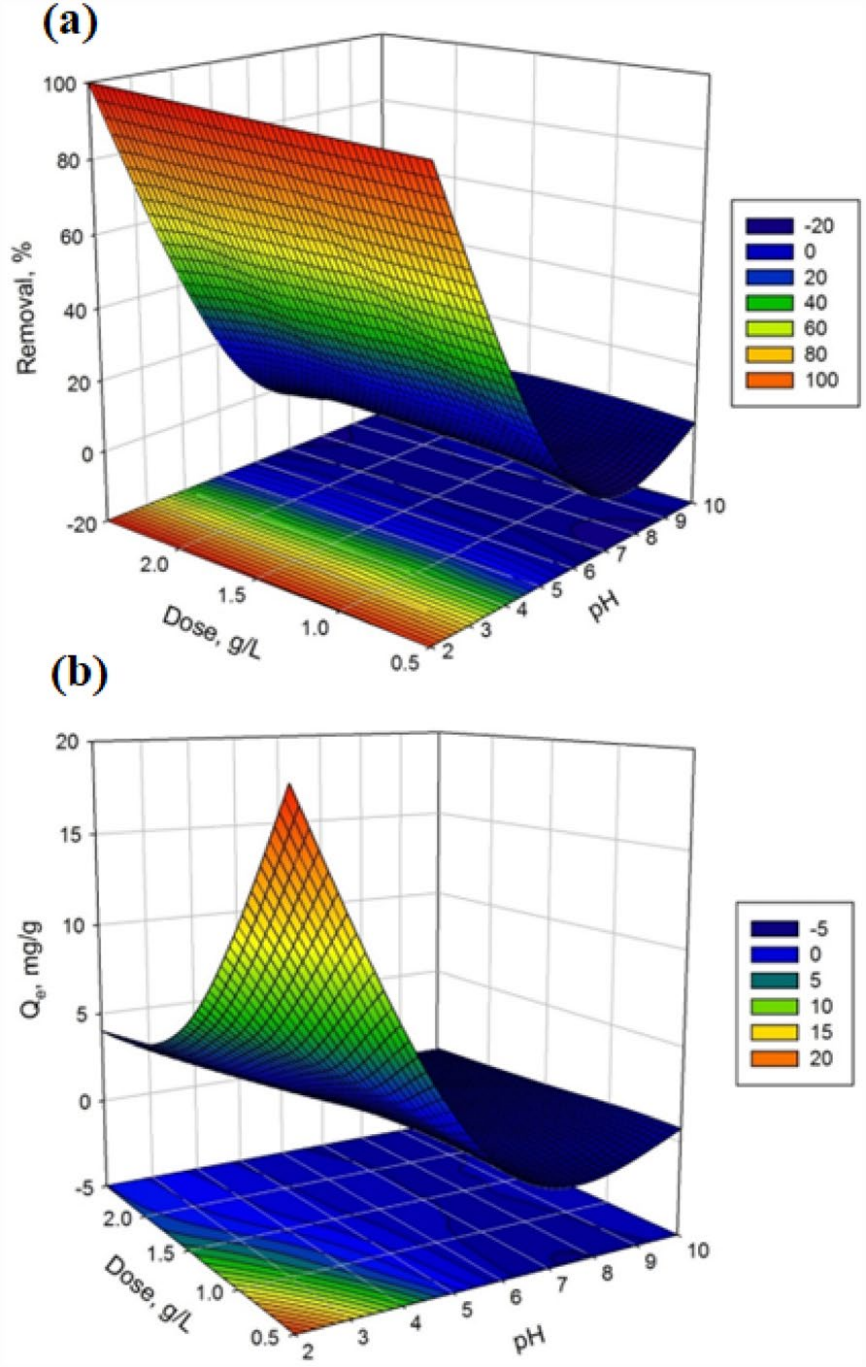
Response surface graphs of (**a**) removal efficiency and (**b**) sorption capacity vs the impact of two variables.

Table 2 and Fig. 5a,b display the experimental design matrix, along with the independent variables, actual and coded values, and the corresponding removal percentages and adsorption capacities. The experimental design matrix provides essential information about the influences of various variables on the capturing efficiency and sorption capacity of the PU@nZVI system. The variables considered in this study include the dosage of the material in grams per liter (g/L) and the pH. According to the obtained results, it can be observed that the removal percentages and adsorption capacities vary across different experimental conditions. For example, in rows 5 and 9, where the pH was set to 2, the removal percentages reached 97.62% and 100%, respectively. These results suggest that a lower pH under these specific conditions led to enhanced removal efficiency.

**Table 2:** Experimental design matrix in terms of actual values of the independent variables.

Nos.	Real Value		Removal (%)	Adsorption capacity (mg/g)
	pH	Dose (g/L)		
1	6	1.5	9.96	0.66
2	10	1.5	4.81	0.32
3	6	1.5	6.22	0.42
4	6	1.5	8.19	0.54
5	2	1.5	97.62	6.53
6	6	1.5	5.26	0.35
7	2	0.5	95.55	19.23
8	10	2.5	0.00	0.00
9	2	2.5	100.00	4.01
10	10	0.5	3.49	0.71
11	6	0.5	2.83	0.56
12	6	2.5	5.16	0.21
13	6	1.5	6.63	0.44

Additionally, the dosage variations in this study did not show a significant impact on the removal percentages and adsorption capacities. Comparing rows 2 and 3, where the dose remained constant at 1.5 g/L, the removal percentages differed (4.81% and 6.22%, respectively), indicating that other factors, such as the pH or experimental conditions, may have influenced the outcomes to a greater extent. In row 7, where the dosage is at 0.5 g/L and pH 2, the removal percentage and adsorption capacity were 95.55% and 19.23 mg/g, respectively. It is worth noting that there are instances where the removal percentages and adsorption capacities were low or even zero, such as in row 8. These results indicate that combining specific variables in those experiments leads to low effective removal or adsorption under the given conditions.

**Figure 6:**
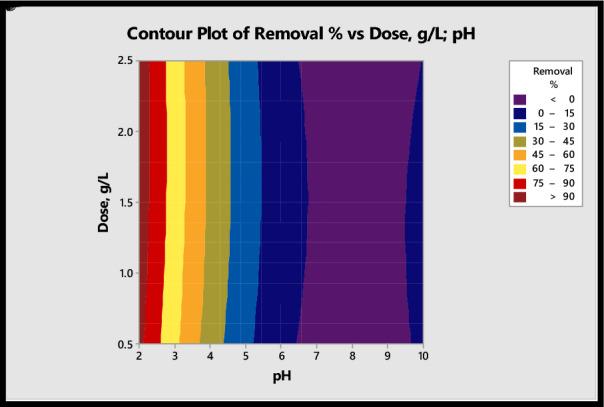
Response surface counterplots of independent variables and removal efficiency of Cr^6+^.

Figure 6 shows the contour graphs of the removal percentage as a function of pH and dose. As concluded, RSM was applied to optimize the two independent variables with the CCD model acquired from experimental data, as discussed before. To achieve removal efficiency > 90%, the optimum predicted values of variables were pH = 2 and initial dose, g/l = 0.50. Implementing the experiments under this optimal condition resulted in a similar removal % (predicted 94.9% and experimental 95.8%), which exhibited the favorability of the CCD model for optimizing the removal of Cr^6+^ onto the PU@nZVI NCs process. In addition, the ANOVA results are shown in Table 3. It could be observed that the pH factor is highly significant in removal efficiency due to the lower p-value (< 0.05). Moreover, pH and dose factors are highly significant in adsorption capacity due to the lower p-value (< 0.05).

**Table 3:** ANOVA analysis of the quadratic polynomial models for Cr^6+^ removal efficiency and adsorption capacity using PU@nZVI NC.

Source	Removal efficiency (%)						Adsorption capacity (mg/g)						
	Sum of squares	df	Mean square	F-value	p-value		Source	Sum of squares	df	Mean square	F-value	p-value	
Model	19,791.58	5	3958.32	1352.74	< 0.0001	Significant	Model	309.25	5	61.85	13.22	0.0019	Significant
A-pH	13,525.73	1	13,525.73	4622.36	< 0.0001		A-pH	137.73	1	137.73	29.43	0.001	
B-Dose	1.8	1	1.8	0.6157	0.4584		B-Dose	44.21	1	44.21	9.45	0.018	
AB	15.77	1	15.77	5.39	0.0533		AB	52.69	1	52.69	11.26	0.0122	
A^2^	5550.95	1	5550.95	1897.01	< 0.0001		A^2^	49.36	1	49.36	10.55	0.0141	
B^2^	15.74	1	15.74	5.38	0.0535		B^2^	3.88	1	3.88	0.8289	0.3929	
Residual	20.48	7	2.93				Residual	32.76	7	4.68			
Lack of fit	6.82	3	2.27	0.6652	0.6158	Not significant	Lack of fit	32.7	3	10.9	733.33	< 0.0001	Significant
Pure error	13.67	4	3.42				Pure error	0.0595	4	0.0149			
Cor total	19,812.06	12					Cor total	342.01	12				

### Adsorption isotherms

The isotherm study is the most appropriate way to design and assess the performance of the sorption process. Fitting experimental equilibrium data gave valuable information about the process. Various models like Langmuir, Freundlich, and Dubinin–Radushkevich (D–R) are used to analyze the adsorption equilibrium data. Adsorption isotherm experiments were carried out in a concentration range from 0.5 to 700 ppm of Cr^6+^ with 0.5 g/L of PU@nZVI NCs at pH 2. Langmuir, Freundlich, and Dubinin–Radushkevich were used to fit the experimental data, which could be expressed in a linear form as illustrated in Equations (S4-8) as shown in Supplementary Information (S1.3.).

**Figure 7:**
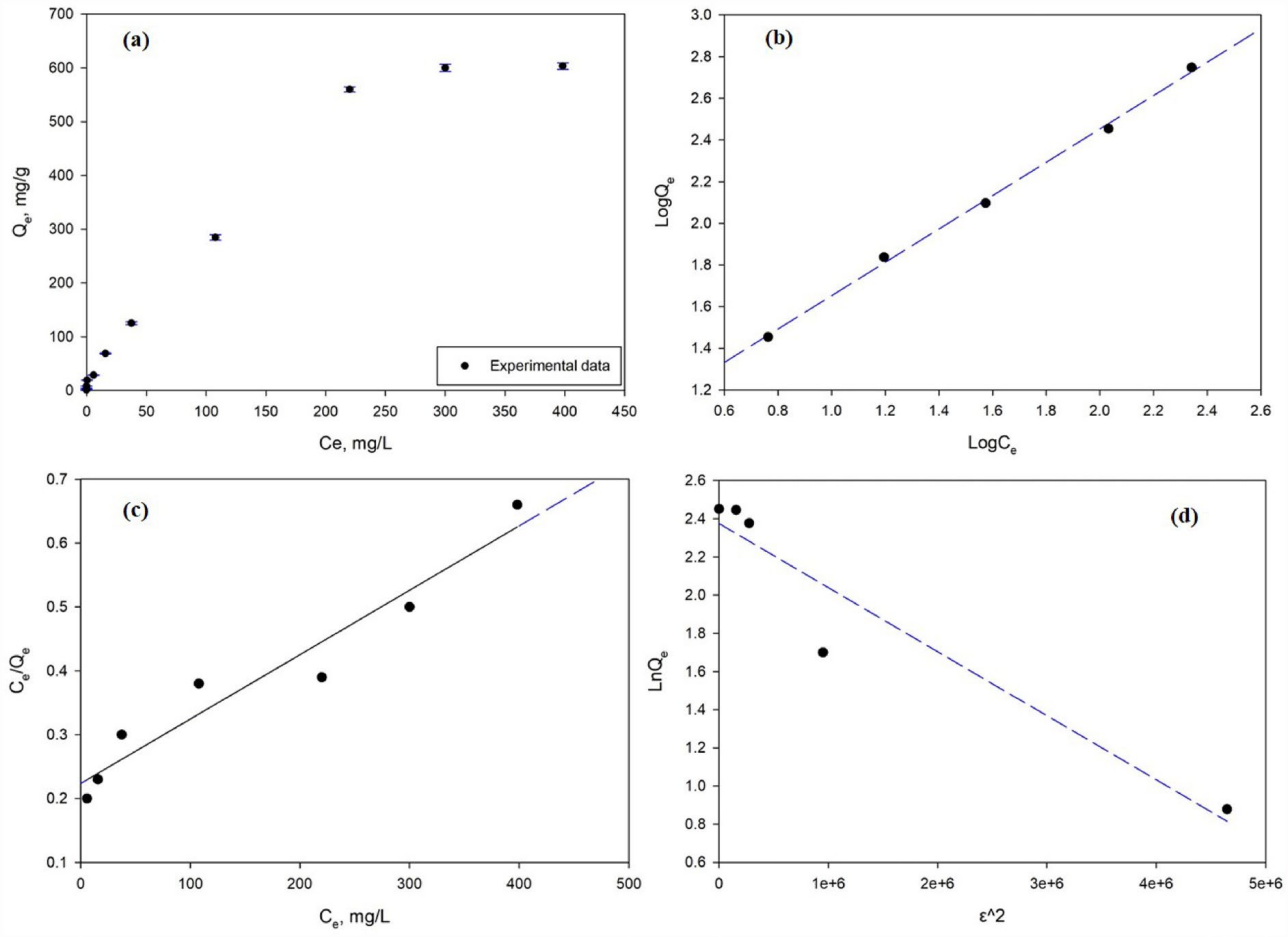
(**a**) Impact of initial Cr^6+^ concentration on the removal of Cr^6+^ by using PU@nZVI CNs adsorbent, (**b**) Freundlich, (**c**) Langmuir, and (**d**) Dubinin-Radushkevich (D-R) isotherms.

The adsorption behaviour for nZVI loaded onto Polyurethane shows that as the concentration of Cr^6+^ rises, the adsorption capabilities of the nanomaterials correspondingly increase. As demonstrated in Fig. 7a, the highest adsorption capability was attained at an equilibrium Cr^6+^ value of 300 mg/L, implying that enhanced adsorption onto the PU@nZVI favours increased Cr^6+^ concentrations. The Freundlich model was more appropriate for characterizing the Cr^6+^ adsorption when the adsorption data was fitted to both the Langmuir and Freundlich models. Figure 7b and Table 4 show the greater regression coefficient (R^2^) found with the Freundlich model, reaching 0.991, which is higher than the value obtained from the Langmuir model (R^2^ = 0.941)^48^.

**Table 4:** Langmuir, Freundlich, and Dubinin–Radushkevich (D–R) isotherm parameters for adsorbing of Cr^6+^ onto PU@nZVI CNs adsorbent.

Langmuir	
Q_m_ (mg/g)	1010.545
K_L_ (L/mg)	0.0044
R^2^	0.941
R_L_	0.961
Freundlich	
K_F_ (mg^1−(1/n)^L^1/n^g^−1^)	8.253
1/n	0.750
R^2^	0.991
D–R	
Q_m_ (mg/g)	559.2
$\beta$ (mol^2^/kJ^2^)	3.00E^−07^
R^2^	0.915
E (kJ/mol)	0.035

Additionally, the value of "n" obtained from the Freundlich isotherm was higher than 3.26, indicating a high affinity between the adsorbate (Cr^6+^) and the adsorbent (PU@nZVI). Also, it further supports the favourable adsorption of Cr^6+^ onto the PU@nZVI, suggesting that the sorption process is primarily physical rather than chemical^49^. Besides, the calculated parameter of energy (E) from Dubinin-Radushkevich isotherm (not provided in the statement) indicated a value of 0.035 kJ/mol for the PU@nZVI CNs adsorbent. This low E value suggests that the interaction between Cr^6+^ and the surface of the nanomaterials is predominantly physical, indicating that the adsorption process requires less energy^48^. The results demonstrate that the adsorption capacity of PU loaded with nZVI for Cr^6+^ increases with higher concentrations of the pollutant.

Langmuir, Freundlich, and D–R isothermal parameters, along with Fig. 7b–d, provide essential ideas into the sorption performance of Cr^6+^ onto nZVI loaded in Polyurethane Foam. These results contribute to understanding the adsorption mechanisms and the efficacy of the studied adsorbents in capturing Cr^6+^ from aquatic media. For instance, the best fitting of Freundlich isotherm proved that multilayer adsorption is the rate-limiting mechanism.

### Kinetic study

**Figure 8:**
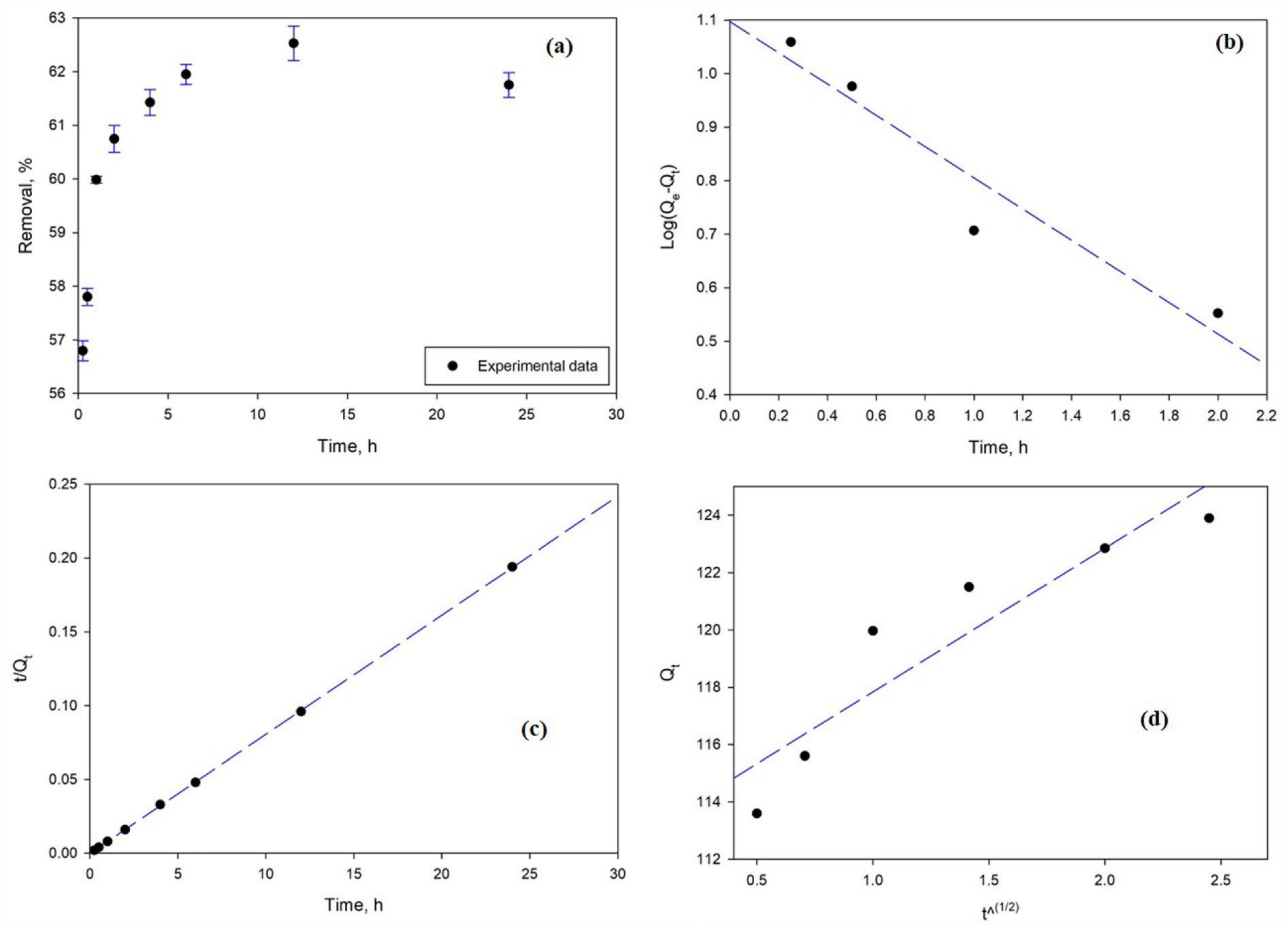
A kinetic study using PU@nZVI (**a**) Influence of contact time on Cr^6+^ adsorption, (**b**) Pseudo-first-order, (**c**) Pseudo-second-order, and (**d**) Intra particle diffusion model.

The adsorption kinetics of PU@nZVI CNs were analyzed by pseudo-first-order, pseudo-second-order, and intra-particle diffusion models^49^ using Equations (S10–12) as shown in Supplementary Information (S1.5.). The effects of removal time on adsorption of Cr^6+^ by using PU@nZVI CNs were investigated (Fig. 8a) under the optimum conditions of pH 2, temperature 298°K and initial Cr^6+^ concentration 100 mg/L, throughout a range of 0.25–24 h. The result showed that adsorption appears relatively fast, reaching equilibrium at about 12 h. Several kinetic models were used to analyze the sorption dynamics, comprising the pseudo-1st-order, pseudo-2nd-order, and intra-particle diffusion models. The pseudo-1st-order kinetic model fails to appropriately characterize the adsorption process, according to the linear graph of log(Q_e_ − Q_t_) against t (Fig. 8b). This is demonstrated by the dissimilarity between the values of calculated Q_e_ (calculated) and Q_exp_ (experimental), indicating that a first-order reaction does not track the sorption of Cr^6+^ on PU@nZVI^48,49^. The values of k_1_ and Q_e_ calculated from the slope and intercept of the graph are presented in Table 5. From the data, the values of Q_e_ (calculated) and Q_e_ (experimental) do not match each other. Consequently, this indicated that the sorption of Cr^6+^ on PU@nZVI was not a 1st-order reaction.

**Table 5:** Kinetics parameters for adsorbing of Cr^6+^ onto PU@nZVI CNs adsorbent.

Pseudo first order	
Q_e_ (mg/g)	12.508
K_1_ (1/min)	0.371
R^2^	0.922
Pseudo second order	
Q_e_ (mg/g)	123.457
K_2_ (g/mg min)	0.656
R^2^	0.9996
Intra particle diffusion	
K_id_ (mg/g/min^1/2^)	5.012
C (mg/g)	112.83
R^2^	0.8573

The findings from experiments, however, and the pseudo-2nd-order kinetic model match well. The pseudo-2nd-order model is appropriate, as shown by the linear connection in the plot of t/qt vs. t (Fig. 8c). The applicability of the model was proved by the greater R^2^ values (Table 5) attained from it as compared to 1st-order kinetics. The fact that the calculated and experimental Q_e_ values correspond supports the argument that the 2nd-order kinetic model describes the removal process by PU@nZVI sorbent. The pseudo-2nd-order kinetic model accurately describes the fast adsorption rate of Cr^6+^ onto PU@nZVI NCs, while the pseudo-1st-order model is inapplicable. These results add to understanding how PU@nZVI behaves as a sorbent used in removing Cr^6+^ by providing valuable insights into the pace and processes of the removal process.

The intra-particle diffusion model treated the experimental data as described in Eq. S12. In this equation, Q_t_ represents the quantity of Cr^6+^ adsorbed at time t (mg/g), K_id_ for the intra-particle diffusion rate constant, and C for the intercept. Intra-particle diffusion plays a central role in governing the kinetics of the sorption route. The intra-particle diffusion model describes how metal ions move from the fluid layer around the adsorbent surface to the interior adsorption sites utilizing pores, where they determine the rate of diffusion. The significant concentration differential between the film and the solid surface causes solute transfer into the surface to occur quickly^50^. Since the Pseudo-2nd order showed the best fitting, the metal ion interaction with the exterior surface of the adsorbent inferred the rate-limiting mechanism^1^.

The thickness of the boundary layer may be calculated by examining the intercept (C) value presented in Eq. S12. Higher intercept values indicate a more significant impact on the boundary layer, but higher K_id_ values indicate a rise in the adsorption rate. Conversely, higher intercept values reflect a more substantial effect on the border layer. The plot of Q_t_ vs. t^(1/2)^ for the earliest stage of Cr^6+^ adsorption on nZVI shows a linear relationship (Fig. 8d). However, the existence of a high C value, as shown in Table 5, indicates that intra-particle diffusion is not the step that determines rate but rather that boundary layer diffusion is somewhat in charge of rate^50^.

### Thermodynamic study

**Figure 9:**
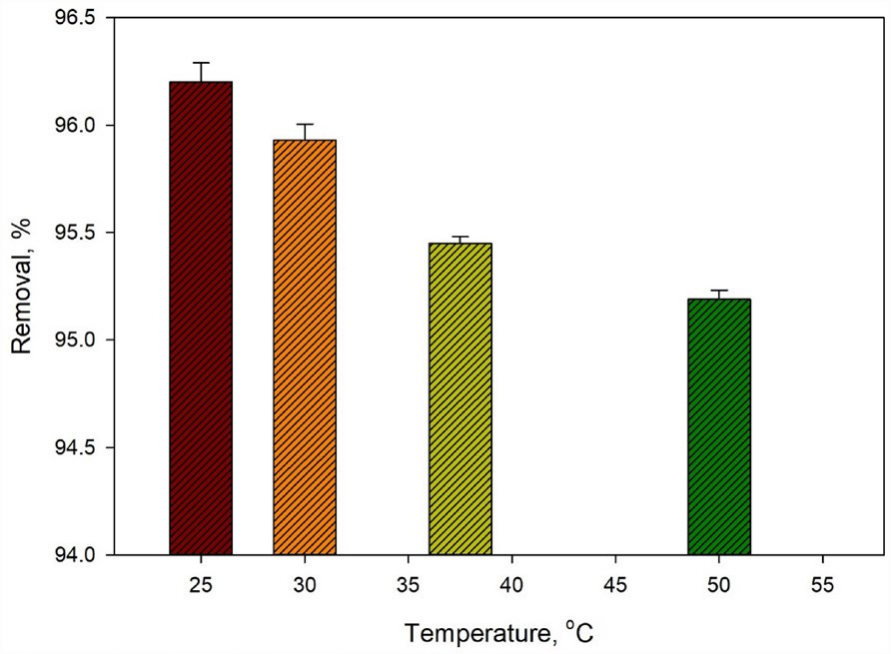
Effect of temperature on Removal % of Cr^6+^.

The thermodynamic study examined the impact of temperature on the sorption percentage of Cr^6+^, as displayed in Fig. 9. Thermodynamic functions, including ∆G°, ∆H°, and ∆S°, were used to analyze the sorption route. The correlation between these functions can be described by the equation ∆G° = ∆H° − T∆S°, where T represents the temperature in Kelvin. Figure 9 provides valuable information about the thermodynamic behavior of the adsorption process and reveals the influence of temperature on the capturing percentage. Higher temperatures can inhibit removal efficiency, while lower temperatures may enhance removal percentages. This study enhances our understanding of the sorption mechanism and the thermodynamic parameters governing the removal of Cr^6+^ using the specific adsorbent studied.

**Figure 10:**
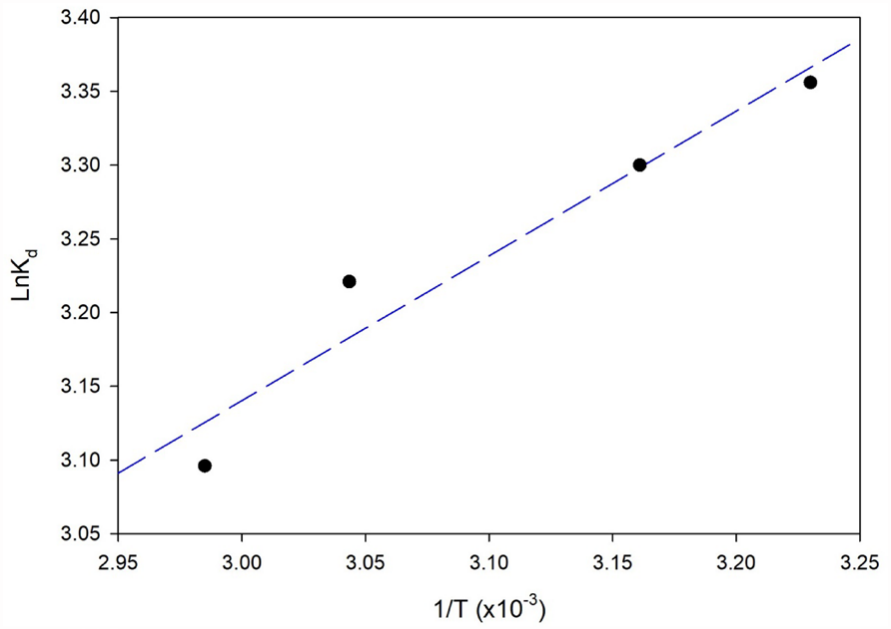
A plot of ln K_d_ vs. the reciprocal temperature for Cr^6+^ removal using nZVI synthesized loading in Polyurethane Foam.

The thermodynamics study investigated the relationship between ln K (natural logarithm of the equilibrium constant) and 1/T (reciprocal temperature) for the removal of Cr^6+^ using nZVI synthesized loading in Polyurethane Foam, as shown in Fig. 10. The equilibrium constant (K) is a fundamental parameter in thermodynamics that indicates the extent and directionality of a chemical reaction at equilibrium. The reciprocal temperature (1/T) is utilized to consider the temperature dependence of the equilibrium constant.

The relationship between ln K and 1/T can be expressed by the Van’t Hoff equation S13 as illustrated in Supplementary Information (S1.5.) ^49,50^.

By plotting ln K against 1/T, Fig. 10 allows for determining thermodynamic parameters and assessing the adsorption process. The slope of the linear plot represents − ∆H°/R, the ratio of the enthalpy change to the gas constant, which provides insights into the energy changes associated with the adsorption process. The intercept of the linear plot represents ∆S°/R, the ratio of the entropy changes to the gas constant that indicates the randomness or disorderliness of the system.

Based on the results presented in Fig. 10, one can analyze the thermodynamic behavior of the adsorption process. The plot allows for determining the enthalpy change (∆H°) and entropy change (∆S°) through the slope and intercept, respectively. These thermodynamic parameters provide information about the energy requirements and the degree of disorder in the adsorption process.

The thermodynamics study presented in Fig. 10 enhances our understanding of the Cr^6+^ removal mechanism using nZVI synthesized loading in Polyurethane Foam by providing valuable insights into the thermodynamic parameters of the adsorption process. It allows for assessing the feasibility and energetics of the adsorption, contributing to the optimization and design of efficient adsorption systems.

The thermodynamic parameters for the nZVI synthesized loading in Polyurethane Foam are presented in Table 6. The negative values of ∆G° (− 7.999, − 7.959, and − 8.012 kJ/mol for 298, 303, and 323 K, respectively) confirm the feasibility of the process was indicative of the favorable and spontaneous nature of adsorption with a high preference for Cr^6+^ onto nZVI loaded onto PU foam, The negative value of the enthalpy, ΔH°, verifies the exothermic nature of the process^49^. The positive entropy, ΔS°, values confirmed the increased randomness at the solid–solute interface during adsorption^48^. Also, the ∆H° value suggests the binding between Cr^6+^ ions and PU@nZVI is a physical adsorption process^48^.

**Table 6:** Thermodynamic parameters for nZVI synthesized loading in Polyurethane Foam.

Temperature (K)	ΔG° (kJ/mol)	ΔS° (kJ mol K)	ΔH° (kJ/mol)	R^2^
298	− 7.999	1.64	− 8.16	0.9368
303	− 7.959			
310.5	− 7.853			
323	− 8.012			

### Regeneration and reusability tests

The results from the experiments indicate that the initial application of PU@nZVI (nano zero-valent iron loaded on Polyurethane) for Cr^6+^ removal in water was highly influential, achieving a removal efficiency of 95.47%. However, the capability of PU@nZVI to eliminate Cr^6+^ gradually decreased with sequential regeneration treatments using different regenerates (HCl, HNO_3_, and NaCl).

**Figure 11:**
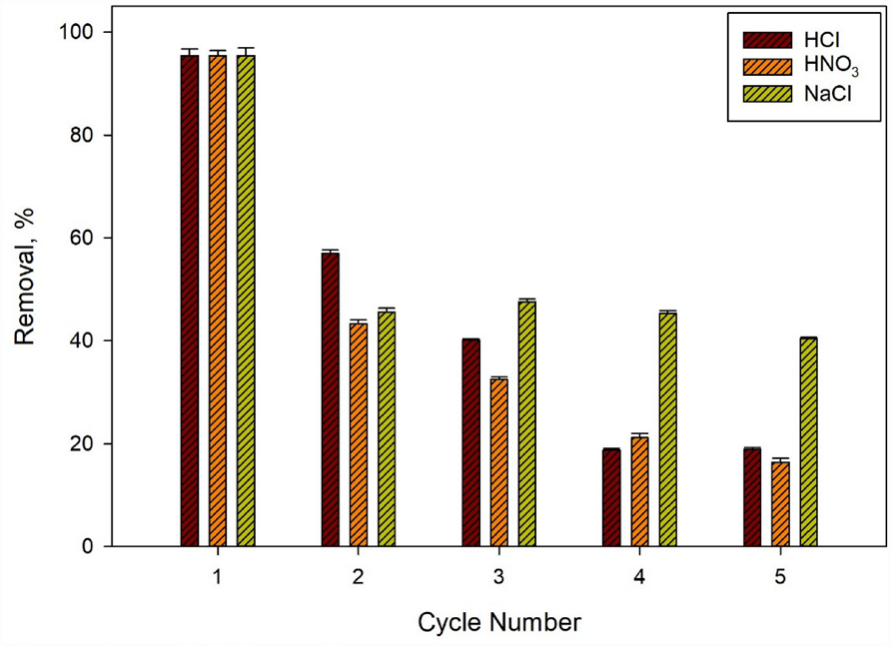
The regeneration-reuse ability of nZVI synthesized loading in Polyurethane Foam in Removal of Cr^6+^.

After two regenerations, the removal efficiencies for HCl, HNO_3_, and NaCl were 57%, 43.36%, and 45.6%, respectively (Fig. 11), demonstrating a significant decrease compared to the initial removal efficiency. This trend continued with further regenerations. After three regenerations, the removal efficiencies dropped to 40.11%, 32.47%, and 47.61% for HCl, HNO_3_, and NaCl, respectively. After four regenerations, the removal efficiencies decreased to 18.75%, 21.14%, and 45.38%, respectively. Finally, after five regenerations, the removal efficiencies were 18.86%, 16.41%, and 40.48%, respectively.

Based on these results, it can be concluded that HCl and HNO_3_ exhibited poor regeneration performance, as their ability to remove Cr^6+^ significantly declined over multiple regeneration cycles. On the other hand, NaCl demonstrated higher regeneration performance, maintaining a relatively stable removal efficiency of 40.48% even after five regenerations. Then, NaCl is more suitable for the regeneration and reuse of PU@nZVI than HCl and HNO_3_. The higher stability of NaCl, compared to the lower stability of HCl and HNO_3,_ implies that NaCl can sustain its effectiveness for a longer duration of regeneration cycles.

### Applications on tap water and wastewater samples

Our removal methodology was applied to capture Cr^6+^ ions in diverse waters, such as industrial effluent (Cement Factory), treated sewage, and tap water (Table 7). Waters were filtered, and drops of HNO_3_ were added to ensure an initial pH below 2. The pH was adjusted to 2, and the initial concentrations of the Cr^6+^ ions were set at 10 mg/L. Tests were next performed to examine the uptake efficacy of Cr^6+^ ions onto the PU@nZVI NCs. The water matrices were analyzed spectrophotometrically by UV–Vis spectroscopy (at 540 nm) to determine the concentrations of Cr^6+^ ions before and after removal. The results showed that the removal percentages of Cr^6+^ from the targeted water samples were 92.45–99.38% by PU@nZVI NCs. As well as the influence of the present optimum condition on the recovery of some anions (as Cl^−^, SO_4_^−2^, and PO_4_^−3^) was conducted, and the results revealed that our composite (PU@nZVI NCs) is efficient in removing these anions, particularly Cl^−^ and PO_4_^−3^.

**Table 7:** The efficiency of nZVI synthesized loading in Polyurethane Foam adsorbents on the Cr^6+^ removal from water.

Matrix	Before adsorption				After adsorption			
	Cr^6+^	Cl^−^	SO_4_^−2^	PO_4_^−3^	Cr^6+^	Cl^−^	SO_4_^−2^	PO_4_^−3^
Tap water	9.761	5.20	10.61	< 0.1	0.002	2.64	9.98	< 0.1
Industrial effluent	8.646	18.51	3.80	29.12	0.276	5.25	3.47	< 0.1
Treated sewage	6.373	36.60	49.10	115.30	0.346	10.98	46.80	18.45

Anions determination was performed using capillary electrophoresis, Agilent 1600CE, Germany.

### Adsorption mechanism

To gain further insight into the adsorption mechanism from the experimental results, the isotherm models, Freundlich and D–R, indicate that the adsorption process followed multilayer adsorption and the mean adsorption energy value is 0.035 kJ/mol suggesting the physical adsorption process. In addition to, ΔH parameter has a negative value confirming the exothermic nature approving the physisorption process.

### Comparison of the Cr^6+^adsorption capacity with other adsorbents in the literature

The comparative study of the synthesized PU@nZVI NC with the other adsorbents reported in the literature is shown in Table 8. It can be observed that our novel PU@nZVI NC demonstrates a high value of the maximum adsorption capacity for Cr^6+^, which is 600 mg/g. Also, it could be used for five adsorption–desorption cycles. However, some other adsorbents in the literature did not demonstrate any reusability studies for Cr^6+^ removal, such as the biochar modified with Mg/Al, iron oxide impregnated with dextrin, and supermagnetic nanoparticles^51–53^. In addition, although the adsorbent of AF-MnP-L1 exhibited its efficiency for nine consecutive adsorption–desorption cycles for Cr^6+^ removal, it has a lower value of the maximum adsorption capacity, which is reported as 34.70 mg/g^13^. Therefore, PU@nZVI is considered a promising adsorbent nanomaterial for the treatment of wastewater contaminated with Cr^6+^.

**Table 8:** Comparison of the current synthesized NC with other adsorbents in the literature.

Adsorbent	Adsorption capacity (mg/g)	References
Woody activated carbon	241.55	^16^
Activated carbon/nZVI	25.00	^25^
Biochar modified with Mg/Al	38.00	^51^
Zeolite coated with magnetite nanoparticles	43.93	^33^
Iron oxide impregnated with dextrin	17.80	^53^
Amine-functionalized zeolite	13.50	^54^
Rice-husk-based activated carbon	56.82	^48^
AF-MnP-L1	34.70	^13^
Supermagnetic nanoparticles	41.00	^52^
PU@nZVI	600.00	Current work

## Conclusions

In conclusion, this study presents the innovative synthesis and application of a PU@nZVI nanocomposite for the efficient removal of chromium(VI) from water. Key findings include the determination of optimal conditions for Cr^6+^ removal, with the maximum adsorption capacity reaching a notable 600.0 mg/g. The nanocomposite showed high efficiency in removing Cr^6+^ from various water types, including tap water, industrial effluent, and treated sewage wastewater, with removal efficiencies of 99.98%, 96.81%, and 94.57%, respectively. Additionally, the PU@nZVI nanocomposite demonstrated effective reusability for up to five adsorption–desorption cycles. These results underscore the PU@nZVI nanocomposite's potential as a highly efficient and sustainable solution for water treatment applications, particularly in chromium(VI) removal. The study contributes significantly to the field by introducing a novel, cost-effective, and high-performing material for environmental remediation.

## Future perspectives in the present study

In the present study, while the PU@nZVI nanocomposite has shown promise in removing chromium (VI), several challenges and future perspectives need to be addressed. One of the main challenges is scaling the synthesis process for industrial applications while maintaining efficiency and cost-effectiveness. Future research could focus on optimizing the production process for large-scale applications. Additionally, long-term stability and potential environmental impacts of the nanocomposite after usage are areas that require further investigation. Exploring the regeneration and disposal methods of used nanocomposites is crucial to ensure environmental safety. Lastly, expanding the application scope to remove other hazardous contaminants could broaden the utility of this technology in water treatment.

### Supplementary Information

Supplementary Information.

## Data Availability

All data generated through this study are included in this manuscript and the Supporting Information file.
