# Supplementary material for: Hexavalent chromium ion removal from wastewater using novel nanocomposite based on the impregnation of zero-valent iron nanoparticles into polyurethane foam
Source: Sci Rep. 2024 Mar 5;14:5387. doi: 10.1038/s41598-024-55803-1 (PMC10914806; doi:10.1038/s41598-024-55803-1)
Supplement: Supplementary file 1 — Supplementary Information. [file 41598_2024_55803_MOESM1_ESM.docx]

**Hexavalent chromium ion removal from wastewater using novel nanocomposite based on the impregnation of Zero-valent Iron nanoparticles into Polyurethane Foam**

**Eman M. Saad^1*^, Mohammed F. Abd-Elhafiz^1^, Eman M. Ahmed^2^, Ahmad Abo Markeb^3^**

*^1^ Chemistry Department, Faculty of Science, Suez University, Suez, Egypt*

*^2^Chemistry Department, Faculty of Engineering, South Vally University, South Vally,Egypt*

*^3^ Department, Faculty of Science, Assiut University,Assiut, Egypt*

*Email:* [*emmsaad@yahoo.com*](mailto:emmsaad@yahoo.com)

**S1. Materials and Methods**

**S1.1. Preparation of solutions**

Hexavalent chromium, Cr^+6^, stock solution (1000 mg/L) was prepared by dissolving an exact quantity of K_2_Cr_2_O_7_ into Milli-Q water containing HNO_3_ acid solution (0.5 %). Working and standard solutions of Cr^+6^ were prepared by appropriate dilution of its stock solution in Milli-Q water. The Diphenylcarbazide (DPC) indicator solution was prepared by dissolving a proper quantity of powdered DPC in acetone. Solutions of HCl or NaOH were used to adjust pH.

**S1.2. Adsorption testing**

$Q_{e}=\frac{\left( C_{o}- C_{e} \right)V}{m}$ …………………………… Eq. S1

$Q_{t}=\frac{\left( C_{o}- C_{t} \right)V}{m}$ …………………………… Eq. S2

$Removal, \% =\left[ \frac{\left( C_{o}- C_{e} \right)}{C_{o}} \right]100$ ………… Eq. S3

C_o_, C_e_, and C_t_ (mg/L) are the Cr^+6^ concentrations initially, at equilibrium and at time t, respectively. V is the initial volume of solution, and m is the weight of PU @nZVI added to the flask.

**S1.3. Isotherm Models**

Langmuir isotherm:

$\frac{C_{e}}{Q_{e}}= \left( \frac{1}{Q_{max}} \right)C_{e}+\frac{1}{Q_{max}K_{L}}$ …………………………………. Eq. S4

Freundlich isotherm:

$Log Q_{e}=\log K_{f}+\frac{1}{n}\log C_{e}$ …………………………………. Eq. S5

Dubinin–Radushkevich isotherm:

$\ln Q_{e}=\ln Q_{m}- \beta\varepsilon^{2}$ ……………………………………… Eq. S6

$\varepsilon= RT\ln\left( 1 +\frac{1}{C_{e}} \right)$ …………………………………. …... Eq. S7

$E = \frac{1}{\sqrt{-2 \beta}}$ ………………………………………………... Eq. S8

Q_e_ (mg/g) and C_e_ (mg/L) are the adsorption capacity and the concentration under equilibrium, respectively. The Q_m_ (mg/g) is the maximum adsorption capacity, and K_L_ (L/mg) is the Langmuir constant. K_F_ and 1/n are the Freundlich constants related to the adsorption capacity and intensity, respectively. $\beta$ is the constant of the sorption energy (mol^2^/J^2^), R is the gas constant (8.31 J/mol K), T is the absolute temperature (K), $\varepsilon$ is a Polanyi potential, and E is the apparent energy of adsorption per molecule of adsorbate (kJ/mol).

**S1.4. Optimization parameters of Cr^+6^** **removal**

The design consists of three levels (low, medium, and high), coded as (-1, 0,+1), and with a full of 13 runs as shown in Table S1. The experimental data were analyzed using Design Expert software (version 6.0.6, STATEASE Inc., USA).

| **Table S1.** Independent variables and levels used in CCD | | | |
| --- | --- | --- | --- |
| **Factors** | **Low (-1)** | **Medium (0)** | **High (+1)** |
| pH | 2 | 6 | 10 |
| Dose, g/L | 0.5 | 1.5 | 2.5 |

$Y= \beta_{0}+ \sum_{i=1}^{k} \beta_{i}X_{i}+\sum_{i=1}^{k} \beta_{ii}X_{i}^{2}+ \sum_{i=1}^{k-1} \sum_{j=2}^{k} \beta_{ij}X_{i}X_{j}$ …………. Eq. S9

Where Y β_0_, β_i_ (i=1, 2, …, k), β_ii_ (i=1, 2, …, k) and β_ij_ (i=1, 2, …, k, j= 1, 2, …, k) are the response, x_i_, x_j_, …., x_k_ are the input variables, the intercept term, the linear effect, the squared effect and the interaction effect, respectively.

**S1.5. Kinetic and thermodynamic equations**

Log (q_e_ –q_t_)= log (q_e_) - $\frac{k1}{2.303}$ t …………………………….. Eq. S10

where q_e_ and q_t_ are adsorption capacity (mg/g) at equilibrium and at time t, respectively, and k_1_ is the rate constant of pseudo-first-order adsorption.

$\frac{t}{qt}=\frac{1}{k2 q2}+\frac{1}{qe}(t)$ ……………………………………... Eq. S11

Where k_2_ is the rate constant of pseudo-second-order adsorption.

Q_t_=k_id_ t^1/2^+C ……………………………………..................... Eq. S12

K_id_ is the intra-particle diffusion rate constant (mg/g. min^−2^).

ln K = -∆H°/R * (1/T) + ∆S°/R ………………………………. Eq. S13

where ∆H° is the standard enthalpy change, ∆S° is the standard entropy change, and R is the universal gas constant.
